# Hydrodynamic behavior and start-up performance of a periodic anaerobic baffled reactor in an “every second” switching manner treating traditional Chinese medicine wastewater

**DOI:** 10.3389/fmicb.2023.1282906

**Published:** 2023-10-27

**Authors:** Xiaolei Liu, Yixing Yuan, Nanqi Ren

**Affiliations:** ^1^School of Water Conservancy and Environment Engineering, Changchun Institute of Technology, Changchun, China; ^2^State Key Laboratory of Urban Water Resource & Environment, Harbin Institute of Technology, Harbin, China

**Keywords:** periodic anaerobic baffled reactor, switching manner, traditional Chinese, computational fluid dynamics, residence time distribution, industrial wastewater treatment

## Abstract

Most studies focus on the “clockwise sequential” switching manner for a four-compartment periodic anaerobic baffled reactor (PABR), while the exploration of the “every second” option on the feasibility for real industrial wastewater treatment is rarely reported. Hence, a PABR-treating traditional Chinese medicine wastewater was run continuously in “every second” switching manner with both switching period T and hydraulic residence time of 48 h. Satisfactory start-up performance was achieved during the operation of a climbing average organic load rate at approximately 1, 2, 4, and 6 kg chemical oxygen demand (COD) m^−3^ d^−1^ for 12, 24, 24, and 6 days, respectively. The average COD removal was 87.20% after the second lifting of OLR and 89.98% after the third one. Denaturing gradient gel electrophoresis and its cluster analysis showed that the microbial communities in each compartment adapted their structure in response to the periodically changing micro-ecology conditions. Moreover, the residence time distribution test with tap water in the clean PABR was carried out in experiments and computational fluid dynamics (CFD) simulation, both of which were in good agreement. The CFD model output visualized the flow velocity field and hydrodynamic-mass transport inside the PABR. Optimization of operation pattern in PABR including switching manner and frequency depended on both the type of waste being treated and the flexibility of biomass to periodically changing micro-ecology conditions.

## 1. Introduction

The traditional Chinese medicine (TCM) industry has become one of the critical industrial systems in China currently because of the unique effectiveness of TCM with a long history. COD discharged from the wastewater of manufactured medicines fluctuated in a range of 3.0–4.2% of that from the total industrial wastewater between 2016 and 2020 (National Bureau of Statistics, 2016, 2017, 2018, 2020). Accordingly, TCM wastewater, generated from pretreatment washing water, drug boiling water, drug juice, and organic solvent and recovered from the drug extraction and concentration process, post-filtration wastewater, post-extraction wastewater and residue, equipment cleaning water, boiler ash and dust removal water, reflux cooling water, and domestic sewage (Huang et al., 2022), accounts for an increasing proportion of total discharge of pharmaceutical wastewater.

TCM wastewater has high levels of organic matter that is difficult to dissolve and precipitate and has high chromaticity, bad odor, and a complex toxic pollution composition (Su et al., 2019). Obviously, it would be a disaster for the environment if TCM manufacturers discharged the wastewater randomly or without proper treatment. The Ministry of Ecology and Environment of China issued the *Discharge standard of water pollutants for pharmaceutical industry Chinese traditional medicine category GB 21906-2008* to prevent TCM wastewater pollution. Although pre-treatment or advanced treatment is needed in order to meet the discharge standard, anaerobic biological treatment is still the core technology in TCM wastewater treatment (Lv et al., 2017).

Anaerobic processes have been widely applied in the treatment of sewage sludge and industrial wastewater over the last few decades. The advantages of anaerobic treatment are excellent organic matter removal, less sludge production, low energy requirement, execution of higher loading rates, and considerable production of biogas (Aziz et al., 2019). The success of new high-rate anaerobic technology has encouraged researchers to extend its application to treat wastewater of a more complex nature (Gaida et al., 2017). A periodic anaerobic baffled reactor (PABR), based on the design concept of the anaerobic baffled reactor (ABR) which has been extensively used and suggested as a promising system (Barber and Stuckey, 1999), was initially developed by Skiadas and Lyberatos (1998).

**Figure 1:**
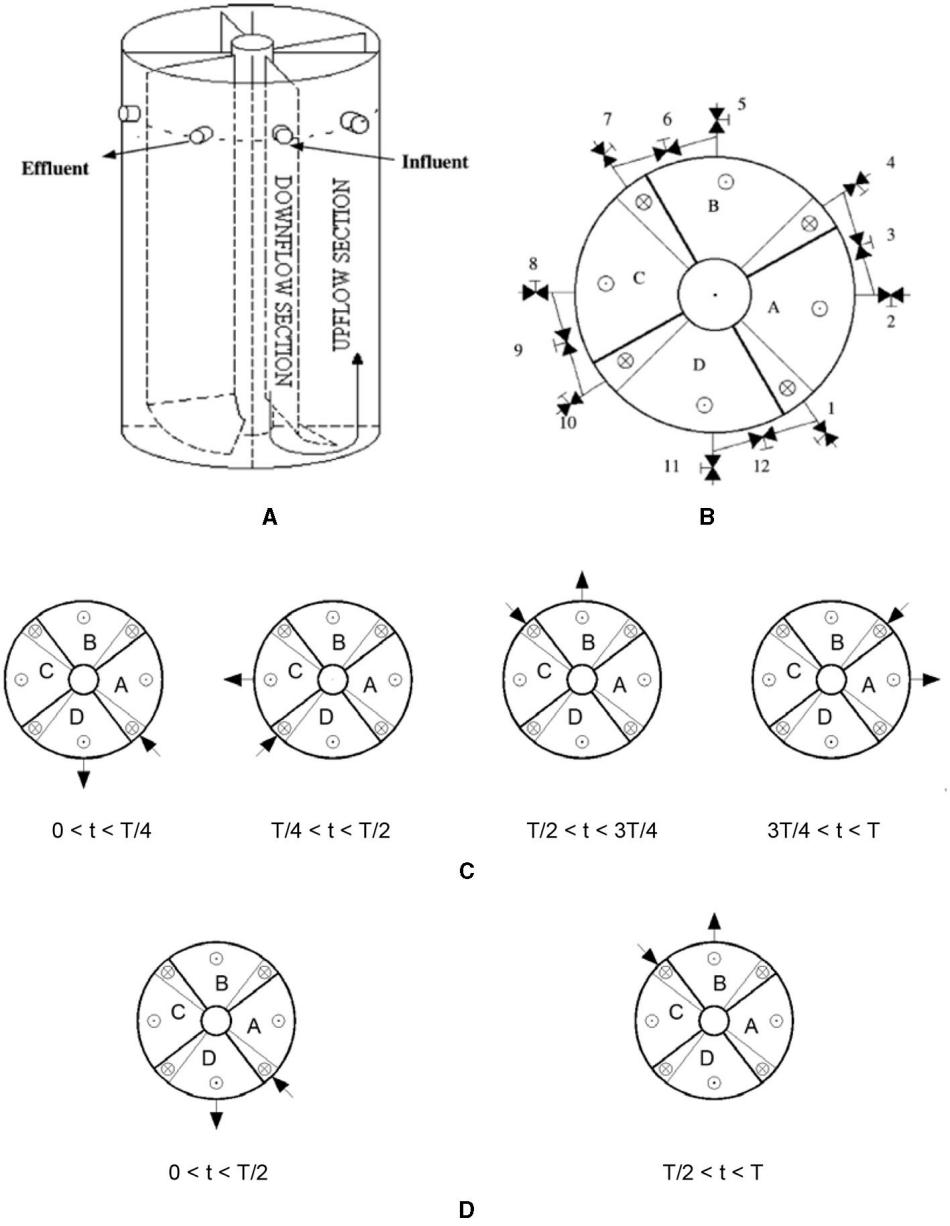
Schematic diagram of a four-compartment PABR. **(A)** Front view. **(B)** Top view: 1; 2; . . .; 12 valves positioned on tubes set outside the reactor. The external tubes connect the up-flow region (⊙) of the compartment with the downflow region (⊗) of the subsequent compartment. **(C)** Order of “clockwise sequential” switching manner. **(D)** Order of “every second” switching manner.

For a four-compartment PABR (Figures 1A, B), there is an influent (feeding) compartment, a second compartment, a third compartment, and an effluent (harvesting) compartment in the sequence of flow pattern. The roles of the four compartments are periodically changed by properly switching (on or off) the 12 valves of the outer tubes. In addition to the switching frequency, there are two switching manners viable for the PABR viewing from the top, namely, “clockwise sequential” (Figure 1C) and “every second” (Figure 1D). Most studies found in the literature focus on the “clockwise sequential” switching manner for synthetic (Skiadas and Lyberatos, 1998; Skiadas et al., 2000; Stamatelatou et al., 2003, 2004), real industrial (Liu et al., 2009; Stamatelatou et al., 2009), or municipal (Zarkaliou et al., 2022) wastewater and food residue biomass (Mathioudakis et al., 2021) treatments, although simulation taken from the study by Skiadas and Lyberatos (1998) predicted that the “every second” option seemed to lead to slightly higher performance and less fluctuation of the effluent concentration compared with the “clockwise sequential”.

As the core of a biological system, the microbial community determines the performance of the bioreactor (Lv et al., 2017). For a PABR, the flexibility to adapt the community structure of biomass in response to periodically changing micro-ecology conditions is the key to degrading pollutants and maintaining the stability of the system. The stability among the individual populations that comprised the microbial community was destroyed by increasing the average organic loading rate (OLR) from 4 to 6 kg COD m^−3^ d^−1^ in a PABR running continuously in a “clockwise sequential” switching manner (Liu et al., 2009). Hence, the feasibility of the “every second” option in PABR for real synthetic or industrial wastewater treatments should be explored to test the prediction mentioned above, especially from the perspective of the microbial community structures in each compartment.

Another important factor leading to a superb performance is the hydrodynamic behavior that affects the mass transport badly in the PABR. A correct description of the mixing behavior of anaerobic digesters is necessary to better predict the system performance (Van Hulle et al., 2014). Unfortunately, to completely understand the hydrodynamic behavior of PABR, discussion about the hydraulic “dead space” and the tank in series model that simulates the mixing pattern through the information provided by experimental residence time distribution (RTD) tests in continuously operating PABR (Liu et al., 2007) may not be sufficient. Therefore, other methods must be used to reveal the visuals inside the PABR equipped with baffles and manifolds in order to clarify how the hydrodynamic behavior affects the performance. Computational fluid dynamics (CFD) has proven to be a helpful tool in the last few years, enabling the simulation of the hydrodynamic behavior of different wastewater treatment systems (Li et al., 2017; Lamhar et al., 2023). The CFD simulation of hydrodynamics in a continuously running PABR was particularly useful for assessing the hydrodynamics in a clean pilot-scale PABR for both the “ABR” operation pattern and the “clockwise sequential” one (Michalopoulos et al., 2018). Therefore, understanding how different operation patterns (combinations of switching manner and frequency) affect the performance is critical to the operation of a PABR. It is important to highlight that CFD studies on a continuously operating PABR in an “every second” switching manner have so far not been published in the literature.

In this study, RTD tests with tap water were carried out both experimentally and with CFD analysis in order to visualize the hydrodynamic-mass transport inside a PABR run continuously in an “every second” switching manner for the first time. Moreover, to compare the “every second” switching manner with the “clockwise sequential” option in start-up performance, handling TCM wastewater for the sake of optimization, TCM wastewater, experimental set-up, seeding, and stepwise increase in OLR were executed, referring to the previous research (Liu et al., 2009). In addition, the flexibility of biomass to periodically changing micro-ecology conditions in PABR was revealed by denaturing gradient gel electrophoresis (DGGE) and its cluster analysis.

## 2. Materials and methods

### 2.1. Experimental set-up

An “every second” switching manner with both switching period T and hydraulic residence time (HRT) of 48 h was implemented on a four-compartment PABR made of Perspex. The diameters of the two concentric cylinders and the height of the PABR with a total useful volume of 18 L were 50, 250, and 500 mm, respectively. In each compartment, the bottom edge of the baffle was set at 20 mm up on the bottom of the reactor, while the volume ratio of the downflow region to the up-flow region was approximately 1:5. Both the inlets of the downflow regions and the outlets of the up-flow regions were placed 100 mm under the top of the reactor, which were connected to the 12 valves of the outer tubes. All the inner diameters of the inlet, the outlet, the valves, and the outer tubes were 10 mm. The temperature in the PABR was maintained at 35°C by a temperature controller, regulating a strip heater twining around the outside cylinder. An ES-B30 Owaki Co. LTD pump was used to supply the flow rate. Biogas collected from each compartment was recorded by a wet gas meter separately.

### 2.2. RTD tests with tap water

#### 2.2.1. Laboratory RTD experiments

The RTD studies were implemented on the clean PABR without inoculation, feeding tap water only, and Li^+^ was employed as a tracer because it did not interact with the walls of the reactor, the valves, or the tubes. An impulse of 10 ml LiCl solution (10 g Li^+^ L^−1^) was injected through a type-Y connector installed before the entrance valve of the reactor when *t* = 0; then, effluent samples were taken right at the exit valve at regularly spaced intervals of 2 h during a period of triple HRT. The concentration of Li^+^ in the effluent was determined using an inductively coupled plasma (ICP) optical emission spectrometer (Optima 5300 DV, PerkinElmer, USA).

#### 2.2.2. CFD simulations of RTD

**Figure 2:**
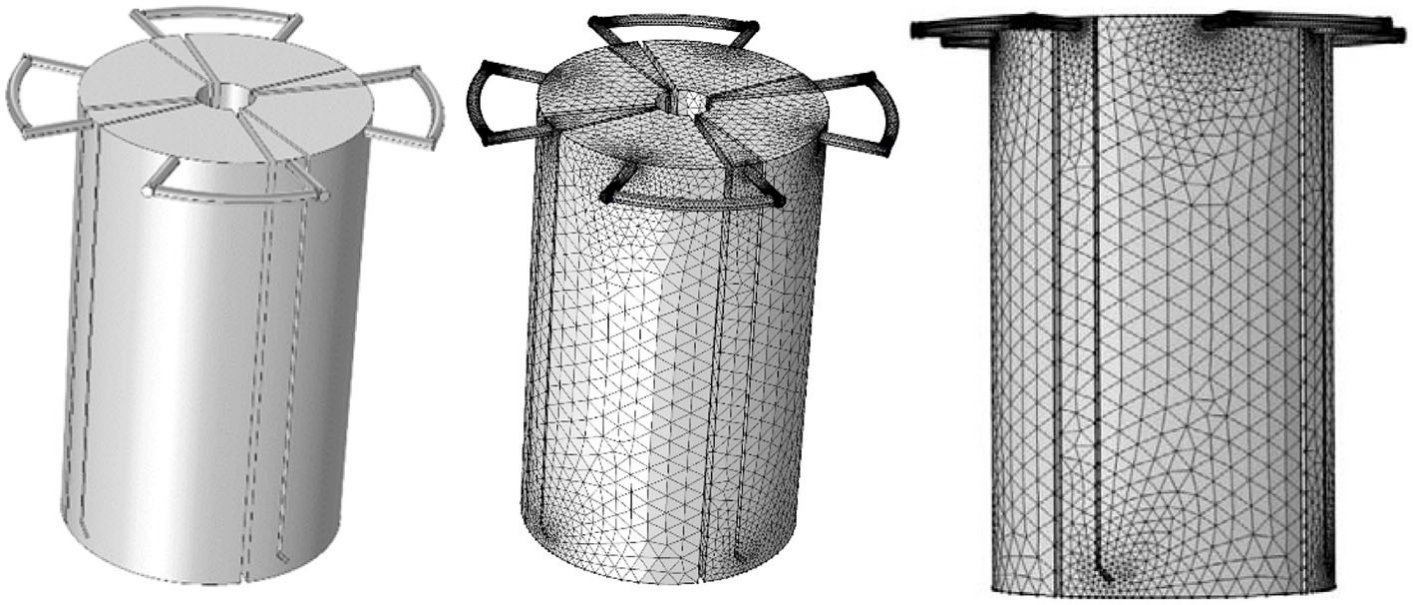
Geometry model of the tap water in PABR and its continuous mesh domain (normal) in an “every second” switching manner.

To simulate RTD curves, tracer diffusion was considered by coupling the Navier–Stokes (N-S) solution to the convection–diffusion equation. Because of the Reynolds number for the flows in the downflow region (Re_*down*_ = 0.6015) and the up-flow region (Re_*up*_ = 0.3638) of each compartment, in the valves and the outer tubes (Re_*v*+*t*_ = 18.352), laminar flow was applied in this study. Under laminar flow conditions at single-phase, N-S equations can describe fluid motion in PABR. Considering incompressible flow and a steady-state regime, N-S equations may be expressed as follows:


(1) $$\begin{array}{l} \rho \left(u\nabla \right)u=\nabla \left[-pI+\mu \left(\nabla u+{\nabla u}^{T}\right)\right]+\rho g \end{array}$$

(2) $$\begin{array}{l} \nabla •u=0 \end{array}$$

where μ denotes the dynamic viscosity of the fluid in Pa · s, *u* is the local component of the velocity vector in m s^−1^, *p* is the pressure in Pa, ρ is the density of the fluid in kg m^−3^, and *I* is the identity vector (Barrios et al., 2019). To work out the solution to the equations, they must be linearized and solved over many small control volumes (the computational mesh). For determinations of the flow field, these simulations require the input of geometry; then, the geometry of the PABR was constructed in 3D using COMSOL Multiphysics software, according to the dimensions mentioned in Section 2.1 (Figure 2). The element size in the mesh settings was normal in order to obtain a good convergence and minimize the computing time. The fluid properties were water (liquid) whose temperature was 308.15 K. On the inlet boundary, the normal velocity of the flow was 1.33 × 10^−3^ m s^−1^. On the outlet boundary, a pressure datum was set along with a zero viscous stress condition, according to the model structure set by Michalopoulos et al. (2018), and the no-slip condition was implemented on the walls of the reactor and the tubes.

The time-dependent behavior of a tracer inside the reactor could be described by the general form of the diffusion–convection equation cited as follows:


(3) $$\begin{array}{l} \frac{\partial c_{i}}{\partial t}=D_{i}\nabla^{2}c_{i}-v\nabla c_{i} \end{array}$$

where *c*_*i*_ is tracer concentration in mol m^−3^, *t* is the time in s, *D*_*i*_ is the tracer diffusion coefficient in m^2^ s^−1^, and *v* is the velocity vector *u* obtained by the solution of equations (1) and (2) for the laminar flow (Barrios et al., 2019). Equation (3) is in a transient state, and therefore, it needs an interval of time to be solved, which was established from t = 0 to t = 24 h in every T/2 = 24 h for the PABR continuously running in “every second” switching manner, at 2 h steps for all calculations with restriction on tracer concentration, except for the first T/2 = 24 h.

Originally, the reactor was filled with water and was fed through one pair of inlet and outlet, while the order of the compartment from the influent to the effluent was A-B-C-D (0 < *t* < T/2) (Figure 1D). The initial concentration of the tracer inside the PABR was 0, and the tracer did not interact with the walls of the compartment, the valves, or the tubes (Michalopoulos et al., 2018). To avoid as much as possible the back-mixing effects at low velocities on the entrance valve where a pulse tracer injection with *c*_0_ = 1,383,285.303 mol m^−3^ was carried out at *t* = 0, the time step for the unsteady state section (concentration change with time) is considered as 0.1 s, which marked as Study 1. Then, Study 2 began with the solution of Study 1 when the order of the compartment from the influent to the effluent was A-B-C-D (0.1 s < *t* < T/2) as the first T/2 = 24 h, except for the 0.1 s of Study 1 at the very beginning. Hence, there were two studies in the first T/2 = 24 h. Next, Study 3 started with the solution of Study 2 when the order of the compartment from the influent to the effluent was C-D-A-B (T/2 < *t* < T) as the second T/2 = 24 h. Then, Study 4 started with the solution of Study 3 when the order of the compartment from the influent to the effluent was A-B-C-D (0 < *t* < T/2) as the third T/2 = 24 h. Thus, the CFD simulations were completed, taking the tracer recovery into consideration till Study 9 ended as the eighth T/2 = 24 h over a period of 4-fold HRT.

The normalized RTD curve should be simulated to compare the fluid flow behaviors in different reactor sizes. E(t), which describes the tracer distribution in certain periods of time for the stream of fluid leaving the reactor, can be assessed according to Eq. (4), cited as follows:


(4) $$\begin{array}{l} E(t)=\frac{C(t)}{\int_{0}^{\infty }C(t)dt} \end{array}$$

where *C(t)* is the time-dependent concentration response evaluated at the exit valve (Rosales et al., 2016), which was achieved by the solution of the Eq. (3). A dimensionless *E(*θ*)* function can be defined as follows:


(5) $$\begin{array}{l} E\left(\theta \right)=HRT•E\left(t\right) \end{array}$$

Moreover, it can be plotted as a function of the dimensionless time θ (= t/HRT). A dimensionless *C(t)/C*_*Average*_ is also required:


(6) $$\begin{array}{l} C_{Average}=\frac{M}{V} \end{array}$$

where *M* is the mass of the tracer injected at *t* = 0, and *V* is the total working volume of PABR.

### 2.3. Sewage and seed

The wastewater was obtained from the same TCM manufacturer located in Harbin city, China, while the digested sludge was obtained from the same brewery, in accordance with the previous article (Liu et al., 2009). Soluble COD and biochemical oxygen demand (BOD) were found to be in the range of 142,407.3–578,368.6 g COD m^−3^ and 71,874.2–295,965.4 g BOD m^−3^, respectively. Moreover, the COD: total nitrogen (TN): total phosphorus (TP) was approximately 226: 5: 1, while the pH value ranged from 6.75 to 7.54. The TCM wastewater was diluted with tap water to feed the PABR during the operation of average OLRs at approximately 1, 2, 4, and 6 kg COD m^−3^ d^−1^ for 12, 24, 24, and 6 days, respectively. In addition, 1.5 NaHCO_3_ g L^−1^ was added to keep the alkalinity and pH of the influent stable. Digested sludge was sieved (0.425 mm) and seeded into each compartment of the PABR at an average biomass concentration of 13.32 g VSS L^−1^.

### 2.4. Sampling and analysis

COD and pH in influent and effluent and biogas production of each compartment were monitored every day. To keep a steady situation for an 18 L PABR as far as possible, the COD and the pH in bulk liquid from each compartment were detected every other day, while the order of the compartment from the influent to the effluent was C-D-A-B (T/2 < *t* < T) (Figure 1D). Samples of bulk liquid from each compartment were taken except for the duration of both days 31 to 36 and days 45 to 50, in line with the previous article (Liu et al., 2009).

The biomass was concentrated in the lower part of the bioreactor due to gravity. It was impossible to take proper homogenous samples to determine the solid concentration in the bioreactor unless the PABR content was removed and collected in a tank to be homogenized. Hence, no sludge sample was taken during the experiment. To measure the mixed liquor volatile suspended solids (VSS), the content in each compartment was removed and collected in a tank to be homogenized at the end of day 66. Biomass samples were collected from each compartment on days 36, 60, and 66, which are the ending days for stages of average OLR at approximately 2, 4, and 6 kg COD m^−3^ d^−1^, respectively.

All items on the quality of the TCM wastewater, the influent, the effluent, and the bulk liquid from each compartment, together with the mixed liquor VSS, were measured according to the standard methods (Rice et al., 2017). DGGE and its cluster analysis were achieved as the same approach taken in the previous article (Liu et al., 2009).

## 3. Results and discussion

### 3.1. Comparison between simulated and experimental RTD curves

**Figure 3:**
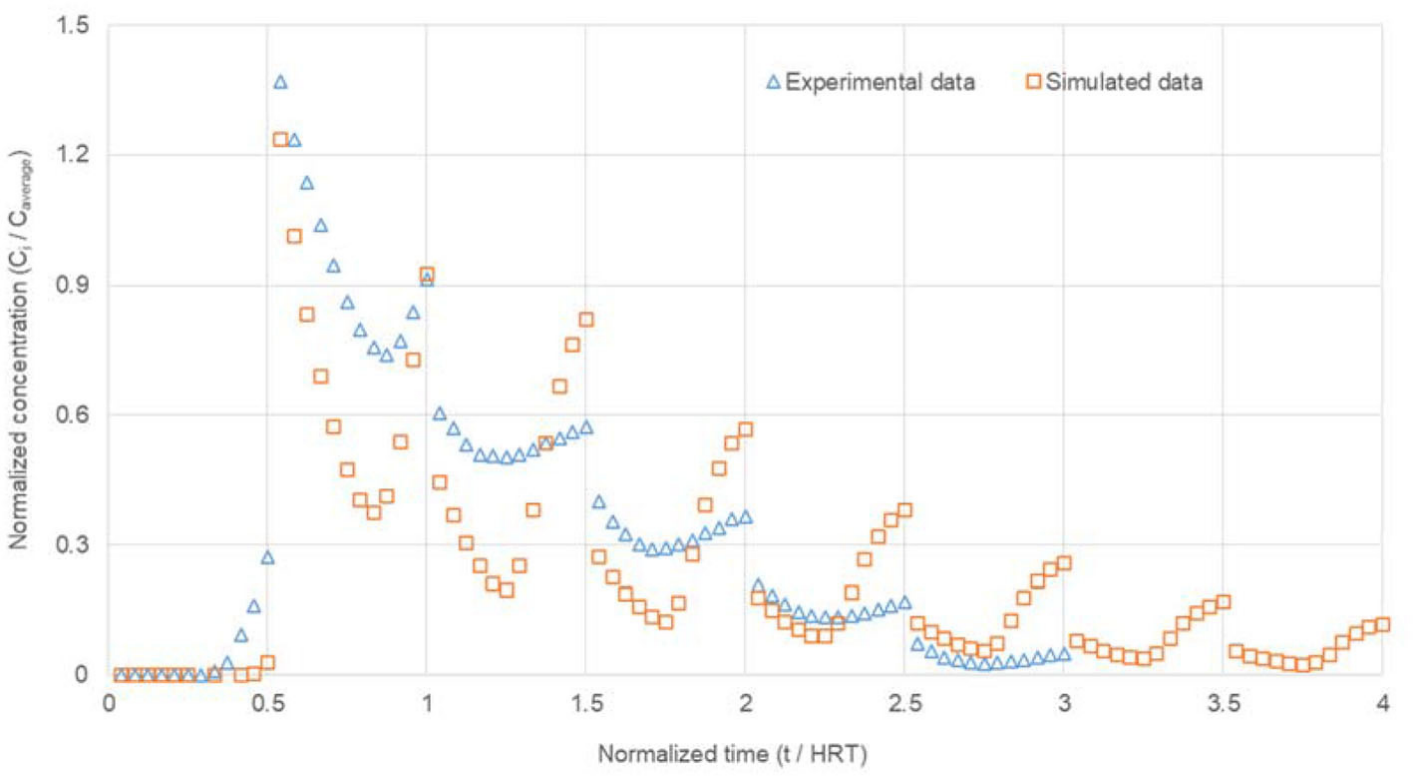
Comparison between simulated and experimental RTD curves for PABR in an “every second” switching manner. Variations for both the horizontal axis [Equation (5)] and the vertical axis [Equation (6)] were dimensionless.

However, during a period of triple HRT, most of the tracer in the RTD experiment recovered (96.34% the tracer recovery in RTD simulations, which climbed up to 94.26% during a period of 4-fold HRT, was only 91.19%, indicating that the pollutants in wastewater had sufficient time to be digested. Hence, the mass balances performed on Li^+^ in both simulated and experimental RTD experiments showed an agreement between the mass injected when *t* = 0 and the mass detected in the effluent during triple HRT for experimental RTD and 4-fold HRT for simulated RTD. Overall, both the simulated and the experimental RTD curves jumped down at the beginning and went up to the end, which resulted in a minimum value of *C(t)/C*_*average*_ during each T/2 = 24 h, except for the first one (Figure 3). Even though there was a deviation, the trend of falling from the second T/2 = 24 h to the last one for the simulated RTD curve agreed with the experimental one. The experimental RTD curve and the simulated one were in good agreement, thus validating the simulation model. Therefore, the results from solutions of N-S for laminar flow and diffusion–convection equations could represent the actual hydrodynamic profile and mass transport of tracer inside the PABR run continuously in “every second” switching manner.

Similar results of deviation between the simulated and the experimental RTD curve were achieved by Michalopoulos et al. (2018) in a clean pilot-scale PABR for both the “ABR” operation pattern and the “clockwise sequential” one. Different geometry, bigger size, and different operations of PABR, whose geometry is really complicated, lead to a different flow. The absolute error arises from several sources, such as numerical solutions, geometry meshing, incomplete iterations, and boundary conditions, and the simplifying assumption has been made (Amani and Jalilnejad, 2017). Further efforts are needed to lower the errors between simulation and ?experimental data for the hydrodynamic study of PABR.

### 3.2. Flow velocity field

**Figure 4:**
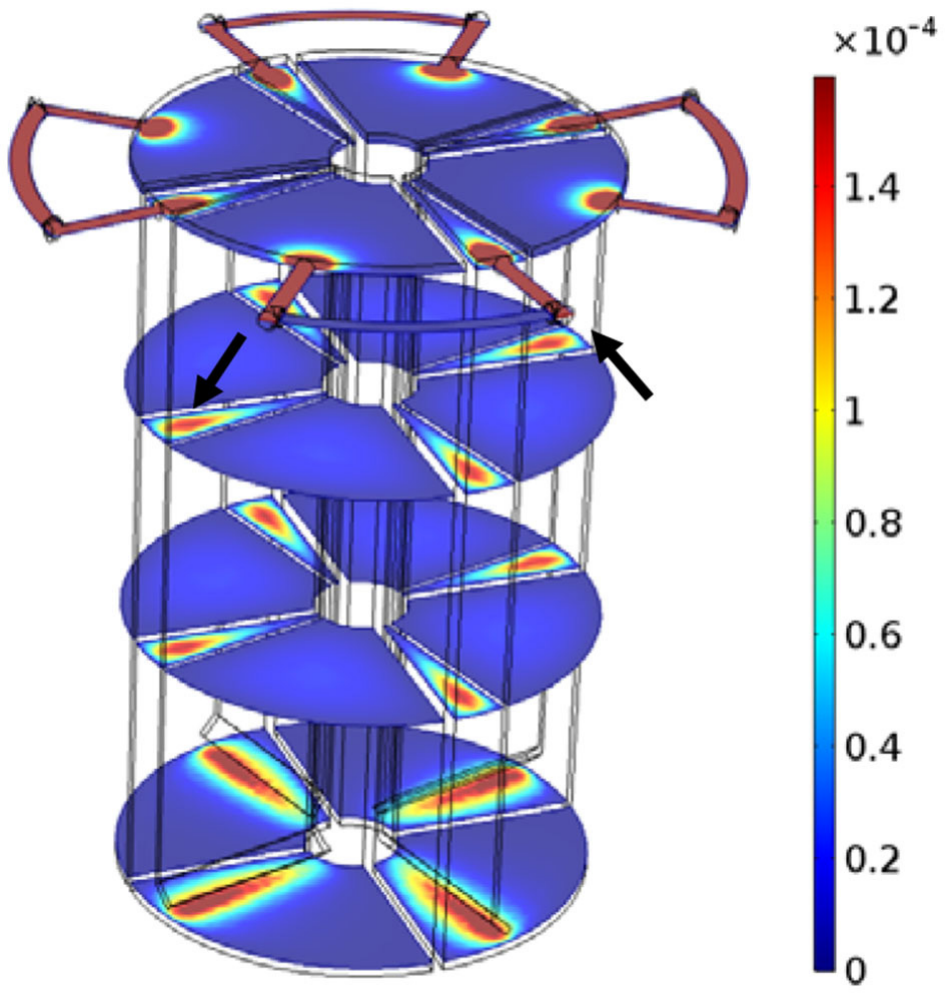
Velocity (m/s) pattern in PABR in an “every second” switching manner.

**Figure 5:**
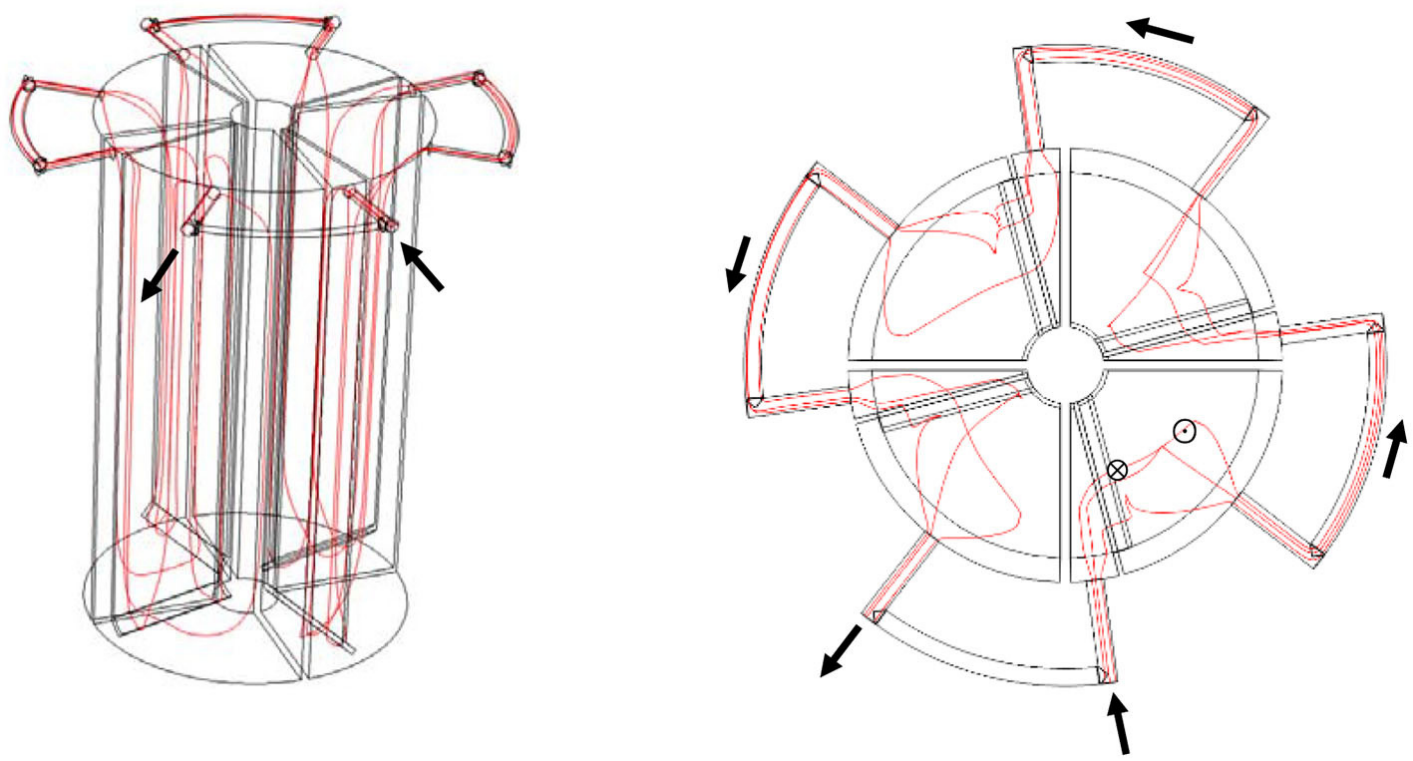
Streamline plots for PABR in an “every second” switching manner.

Figure 4 shows the flow velocity field, which is extracted from the CFD model output, of the fluid in a steady state inside the PABR at a volumetric flow rate of 6.25 ml min^−1^. The high-velocity zone is identified by the red color located in the inlet, the outlet, the downflow region, the region near the bottom edge of the baffle in each compartment, and in the outer tubing, while the low-velocity zone (dark blue color) is presented in the high proportion of the reactor volume which is the up-flow region of each compartment. The average upward velocity rate in the up-flow region, which is the reaction region for the degradation of pollutants, of each compartment was only 0.038 m/h. Lighter blue toward the center of the up-flow region in all four cross-sections at different heights of the PABR verified the hydrodynamic characteristics of laminar flow. As shown in Figure 5, the streamlines were more intense where the flow velocity was high. Obviously, CFD simulations can provide a detailed knowledge of the velocity flow distribution inside the PABR.

### 3.3. Hydrodynamic-mass transport model

Since a satisfactory validation of the hydrodynamic-mass transport model was obtained, variation of the concentration (mol Li^+^ m^−3^) fields was performed in PABR run continuously in “every second” switching manner during a period of 4-fold HRT, as shown in Table 1. During each T/2 = 24 h except for the first one, concentration fields of the first moment (2 h), the moment when *C(t)/C*_*average*_ for the simulated RTD curve was lowest and the last moment (24 h), were displayed through the four cross-sections at different heights of the PABR. The tracer went preferentially through the reactor following the streamline plots (Figure 5), leading to a reduction in the concentration magnitude of the multicolor bar for each T/2 = 24 h in sequence. The tracer transported more slowly where the flow velocity was low, and in the middle two cross-sections of the up-flow region, darker blue toward the center verified a convection-diffusion occurring in accordance with the hydrodynamic characteristic of laminar flow. The concentration-time graphs present the response of the system to a pulse input on the feed and can be used for the prediction of the behavior of the reactor (Michalopoulos et al., 2018). Since compartment A or C took the role as feeding compartment only during a half of T, the input of the tracer was shared periodically by compartments A and C. Then, during the other half of T, it allowed the compartment A or C to alleviate the stress as the third compartment until it was time to become the feeding one again.

**Table 1:**
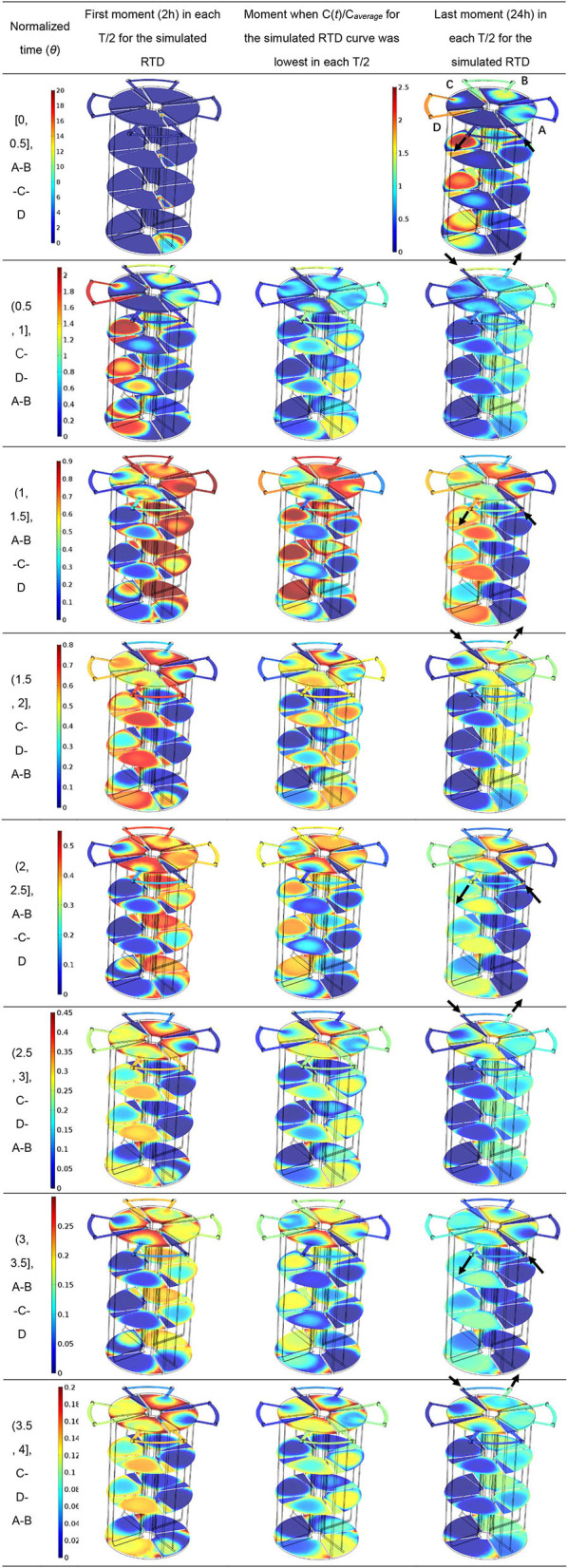
Variation of the concentration (mol Li^+^/m^3^) fields in PABR run in “every second” switching manner.

The multicolor bar located beside images for each T/2 = 24 h represents the magnitude of the concentration field.

The stagnant zones of mass transport evolved in the corner of the up-flow region in each compartment due to the back-mixing effects, which could be found in the bottom cross-section. For the top cross-section, back-mixing effects also caused stagnant zones in external tubes because of the periodic switching of valves, but there was a lighter concentration of the tracer toward the exit valve for the effluent compartment. Focusing on simplifying PABR's geometry is crucial because it affects the dead space percentage, and the CFD simulations can be used for the optimization of the reactor's geometry (Michalopoulos et al., 2018). Thus, the geometry of the PABR should be optimized to scale down stagnant zones in order to reduce the need for preliminary experimental tests, checking the feasibility of new operation patterns for optimization.

### 3.4. Start-up performance of the PABR

**Figure 6:**
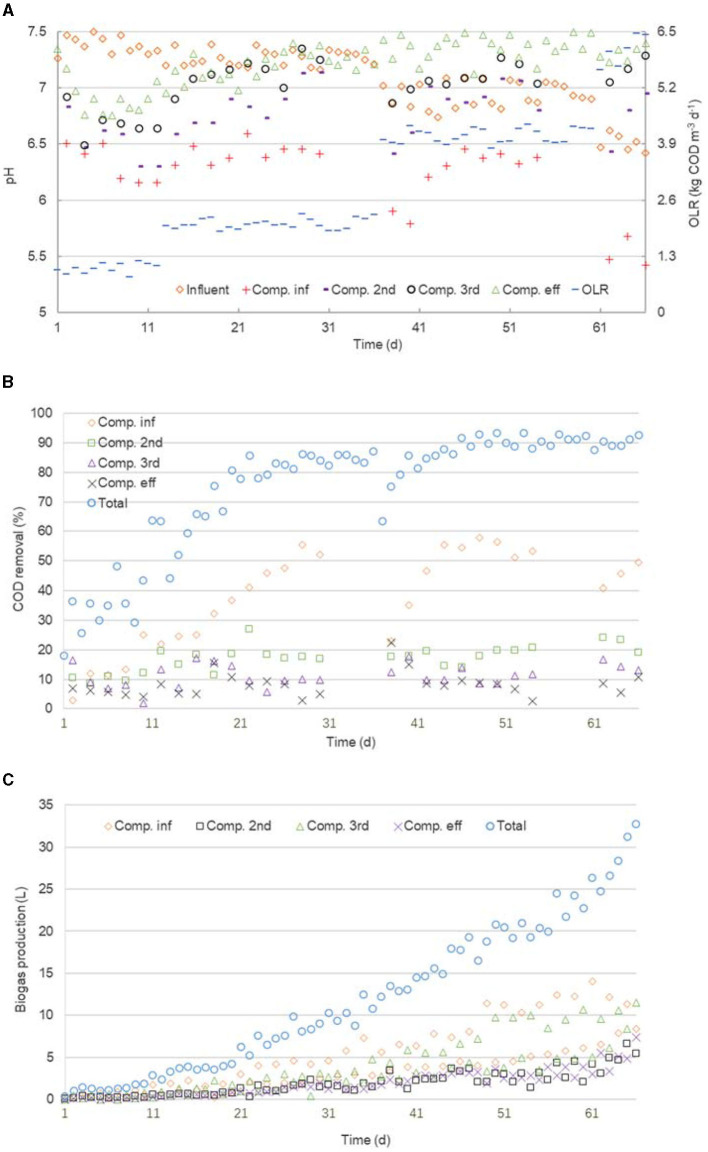
Performance of the PABR in an “every second” switching manner. There is an influent compartment, a second compartment, a third compartment, and an effluent compartment in the sequence of flow pattern. Variation of the pH in influent and bulk liquid (20 mm under the outlet) for each compartment, and of the OLR **(A)**, of the COD removal for each compartment and the total PABR **(B)**, and of the biogas production for each compartment and the total PABR **(C)**.

The PABR was subjected to three successive changes in average OLR from 1 to 2, 2 to 4, and 4 to 6 kg COD m^−3^ d^−1^ by increasing the feed concentration (Figure 6A). Approximately 1.5 g NaHCO_3_ L^−1^ was added to the feed to adjust the pH in the influent compartment, which corresponded to a pH in the feed at normal levels between 6.4 and 7.5 (Figure 6A). The PABR showed, as expected, a transient oscillation of the total COD removal efficiency in every transition (Figure 6B), but the oscillation became smaller as OLR increased. The average COD removal rate was 87.20% after the second lifting of OLR for 24 days and 89.98% after the third lifting for 6 days. In addition, the pH of the effluent compartment was mostly above 7 (Figure 6A), and the total gas production by day increased (Figure 6C) as the OLR went up. Therefore, the PABR tended to be stable. There was no signal that the digestor was going sour in the third transition, compared with an irreversible state of the sharp falls in biogas production, COD removal, and pH of all the compartments for a “clockwise sequential” (T/4 = 24 h) running PABR also treating TMC wastewater found by Liu et al. (2009). In addition, the “every second” (T/2 = 24 h) achieved a higher COD removal of approximately 10% on average during the first two transitions. Thus, for PABR treating TCM wastewater, the “every second” switching option (T/2 = 24 h) was more promising.

The COD removal decreased (Figure 6B) while the pH increased (Figure 6A) in the sequence of flow patterns from the feeding compartment to the harvesting one. Among the four compartments, the pH in the influent one, which contributed most to the total COD removal, obviously dropped. Hence, most pollutants in the TCM wastewater began to be mineralized or even removed in the feeding compartment, whose result was the same as the “clockwise sequential” (T/4 = 24 h) option during the first two successive changes in OLR (Liu et al., 2009).

Owing to the discussion about the stress of the pulse input of the tracer in Section 3.3 (Table 1), a part of complex pollutants in the TCM wastewater within compartment A or C was only digested into intermediates during half of T as the feeding one but was continued to be metabolized into biogas together with the intermediates that came from the previous compartment during the other half of T as the third one. Thus, the biogas production by day in compartment A was similar to that in compartment C as OLR went up (Figure 6C). Therefore, a similar trend of microbial community structure during the whole operation was expected in both compartments A and C. The same conjecture could be made in both compartments B and D.

It was obvious that COD concentration in each compartment declined in the sequence of flow pattern from the feeding to the harvesting, although the COD removal of each compartment decreased. Hence, the biogas production of both the influent (A or C) and the third (C or A) compartments became greater than that of both the second (B or D) and the effluent compartments (D or B) because of getting more substrates. In addition, no granular particle, but only floc sludge, was found in the PABR during the whole operation. Unlike suspended systems such as the UASB, granulation is not necessary in the ABR for optimum performance, and floc size was a function of both biogas production and COD concentration (Barber and Stuckey, 1999). Accumulative biogas mixing in compartment A or C caused the transportation of floc sludge to the next compartment, resulting in the average biomass concentration of compartments A, B, C, and D at the end of day 66 was 14.13 g VSS L^−1^, 15.32 g VSS L^−1^, 14.06 g VSS L^−1^, and 15.98 g VSS L^−1^, respectively. From this point of view, “every second” switching manner enhanced solid retention in compartment A or C to some extent.

### 3.5. Community analysis

**Figure 7:**
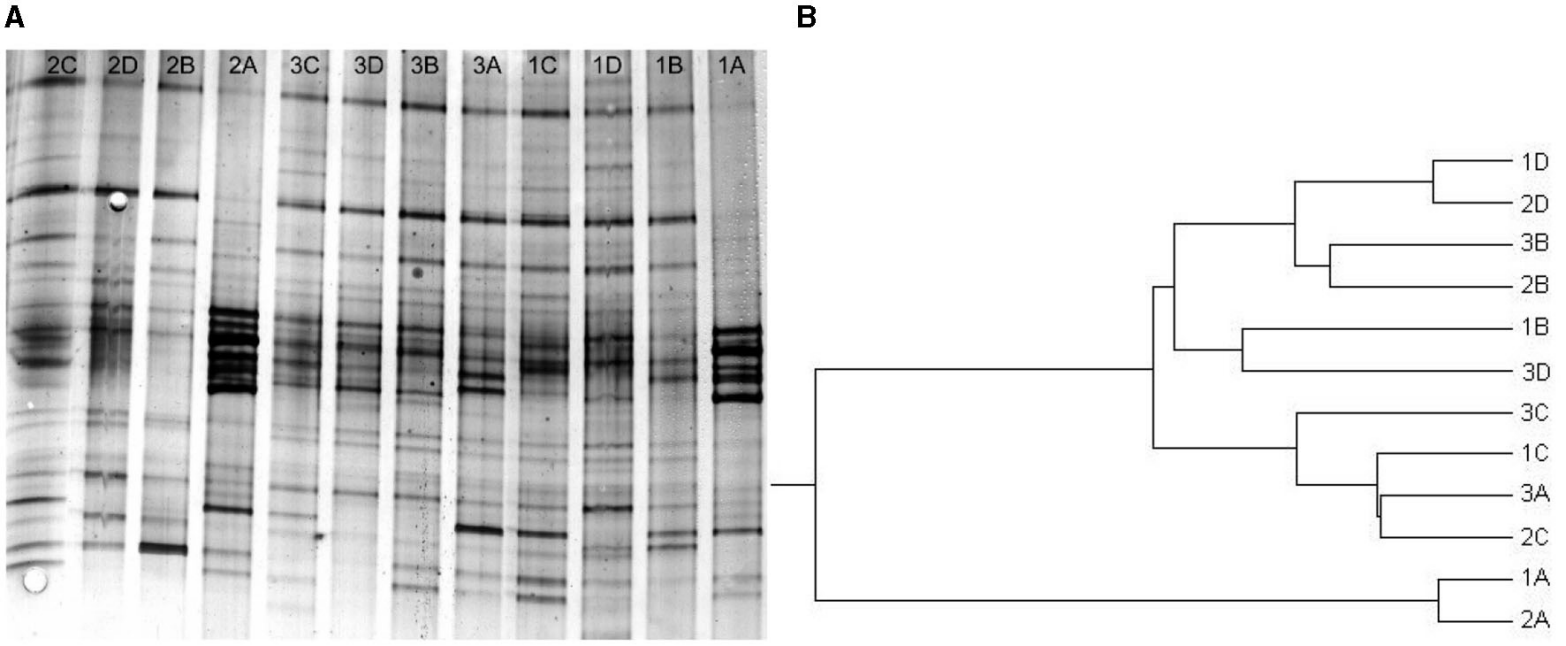
Community analysis of the PABR in an ‘every second' switching manner. DGGE fingerprints **(A)** from each compartment (A, B, C, and D) of PABR at different OLRs, and dendrogram **(B)** revealing the relatedness of DGGE fingerprints, on day 36 at OLR = 2.18 kg COD m^−3^ d^−1^ (1A−1D), day 60 at OLR = 4.09 kg COD m^−3^ d^−1^ (2A−2D), and day 66 at OLR = 6.17 kg COD m^−3^ d^−1^ (3A−3D).

With increasing OLR step-by-step from the initiation (1A−1D on day 36, 2A−2D on day 60, and 3A−3D on day 66), DGGE community fingerprints observed in sludge samples were different from compartment to compartment on the same date (i.e., band numbers and intensity) and from date to date within one compartment (i.e., band appearance and disappearance) (Figure 7A). To get general patterns of community similarity, cluster analysis was performed with the unweighted pair group method algorithm (UPGMA, Figure 7B). Microbial community structures in compartments B and D tended to be more closely related, while those in compartments A and C were more similar, except that sludge samples on day 36 (1A) and day 60 (2A) from compartment A were more distantly related to the others. Therefore, the surmise mentioned in Section 3.4 proved correct. Obviously, for the stable performance of PABR, the individual populations that comprised the microbial community adapted the community structure in response to the periodically changing micro-ecology conditions in each compartment. Hence, the “every second” switching manner allowed the biomass in PABR to withstand the stress caused by lifting OLR of TCM wastewater and to adjust to the new conditions easily during all three transitions. Moreover, interactions between microbial communities require further investigation.

### 3.6. Optimization of operation pattern for PABR

The primary goal of biological wastewater treatment is to sustain optimum reactor performance over long-term operation, even under varying influent wastewater characteristics. According to Skiadas and Lyberatos (1998), the PABR is best suited for handling time-varying loading rates since it always allows for maximal conversion rates. Shortening T could probably improve the PABR performance, as it would result in smaller but more frequent feeding intervals (Skiadas et al., 2000). In addition, PABR functions either as a plug flow in low values of HRT/T or as a CSTR in high HRT/T values (Michalopoulos et al., 2018). Those obtained in previous studies are supported by simulation results for a PABR run in a “clockwise sequential” switching manner. For a giving switching manner, if the influent has high levels of toxic material, or high loading rates are preferred, shortening of T will be beneficial because the exposure of the microorganisms to a severe situation for a prolonged time can enhance the inhibitory effect of the acidic fermentation products on them and limit the rate of their recovery.

In addition to the switching frequency, switching manner is another key factor in optimizing the performance of PABR. The findings of this study showed that in the case of PABR treating TCM wastewater if the biomass did not suffer from too many changes in micro-ecology conditions during one T, the PABR could adjust better to the periodically changing conditions and perform a faster recovery to hydraulic or organic shocks. In contrast, “every second” (T/2 = 24 h) outperformed “clockwise sequential” (T/4 = 24 h) for PABR treating TCM wastewater; though during every 4 days, compartment A or C for “every second” (T/2 = 24 h) undertook feeding compartment twice while each compartment for “clockwise sequential” (T/4 = 24 h) took on feeding compartment only once. In this perspective, the average OLR of 6 kg COD m^−3^ d^−1^ was too high for “clockwise sequential” (T/4 = 24 h) because the biomass in each compartment was subjected to four periodically changing micro-ecology conditions (Liu et al., 2009). However, there were only two periodically changing micro-ecology conditions for “every second” (T/2 = 24 h). Stable performance of PABR over a long-term period required at least some degree of stability among the individual populations in each compartment, and flexibility of the community structure in response to periodically changing micro-ecology conditions was needed.

## 4. Conclusion

For treating TCM wastewater at the same HRT of 48 h, an ‘every second' (T/2 = 24 h) switching manner caused four-compartment PABR stable during the operation of three successive changes in average OLR from 1 to 2, 2 to 4, and 4 to 6 kg COD m^−3^ d^−1^. The average COD removal was 87.20% after the second lifting of OLR for 24 days and 89.98% after the third lifting for 6 days. The “every second” was more promising than the “clockwise sequential” (T/4 = 24 h) manner for TCM wastewater treatment in PABR. DGGE and its cluster analysis showed that the microbial communities in each compartment adapted their structure in response to the periodically changing micro-ecology conditions. The experimental RTD curves and the CFD simulated ones were in good agreement, resulting in successful modeling. A proper manipulation of the hydrodynamic-mass transport data obtained from N-S for laminar flow and diffusion-convection equations simulations on the platform of COMSOL Multiphysics provided flow velocity field and mass transport data detecting the stagnant zones and back-mixing effects, indicating the geometry of the PABR should be optimized. Operation patterns including switching manner and frequency should be optimized depending on both the type of waste being treated and the flexibility of biomass to periodically changing micro-ecology conditions in PABR.

## Data availability statement

The original contributions presented in the study are included in the article/supplementary material, further inquiries can be directed to the corresponding author/s.

## Author contributions

XL: Investigation, Methodology, Writing—original draft, Writing—review and editing. YY: Funding acquisition, Resources, Writing—review and editing. NR: Conceptualization, Project administration, Writing—review and editing.
